# Intracardiac metastasis of gestational choriocarcinoma: a case report and literature review

**DOI:** 10.1186/s12884-023-06144-w

**Published:** 2024-01-02

**Authors:** Yu Gu, Haoran Zheng, Xiaowei Xue, Dan Wang, Hongyan Cheng, Liju Zong, Yang Xiang

**Affiliations:** 1 Department of Obstetrics and Gynecology, Peking Union Medical College Hospital, Chinese Academy of Medical Sciences, National Clinical Research Center for Obstetric & Gynecologic Diseases, Beijing, China; 2 Department of Pathology, Peking Union Medical College Hospital, Chinese Academy of Medical Sciences, Beijing, China No. 1 Shuaifuyuan Road, Dongcheng District, 100730

**Keywords:** Gestational choriocarcinoma, Metastasis, Heart

## Abstract

**Introduction:**

Gestational trophoblastic neoplasia (GTN) with intracardiac metastasis is rare, and here we reported a patient with intracardiac metastasis of high-risk and refractory gestational choriocarcinoma and reviewed relevant literatures.

**Case presentation:**

A 37-year-old woman presented with vaginal bleeding and high level of β-human chorionic gonadotropin (β-hCG) at 199,060 (mIU/mL). It was clinically diagnosed with gestational choriocarcinoma. The patient initially received eight cycles of chemotherapy but unsatisfactory response was observed, and the level of β-hCG still ranged between 5000 and 10,000. Then there was found intracardiac masses in the right atrium (2.6*1.7 cm), anterior chordae tendineae of the tricuspid valve (1.4*0.7 cm) and the right ventricle (4.1*2.9 cm) by ultrasonic cardiogram (UCG). PET/CT highly suspected the intracardiac metastasis of choriocarcinoma (SUVmax = 9.3) and no disease was found in the lung and pelvis. The patient undertook complete intracardiac masses resection. The pathology confirmed the intracardiac metastasis of disease. After a week of operation, the UCG found a 5.4*4.2 cm mass in the right atrium again. Considering the poor prognosis, the patient received palliative care and eventually died of disease progression.

**Conclusion:**

Intracardiac metastasis of GTN is an aggressive sign of disease. Patients can benefit from chemotherapy and surgery. Future investigation of PD-1 immunotherapy combines with chemotherapy are expected to improve the prognosis in this group of patients.

## Introduction

Gestational trophoblastic neoplasia (GTN) is a group of pregnancy-associated malignancies, consisting of gestational choriocarcinoma, invasive mole, epithelioid trophoblastic tumor, and placental site trophoblastic tumor [1]. It originates from malignant proliferation of placental trophoblasts, and the plasm level of β-human chorionic gonadotropin (β-hCG) is the main tumor marker, which is extremely useful for early diagnosis, assessment of treatment response, and prognosis prediction [2, 3]. Chemotherapy is the standard treatment for patients with choriocarcinoma, and the choice of regimen is based on the International Federation of Gynecology and Obstetrics (FIGO) Prognostic Scoring System [4]. Patients at low risk (FIGO score of six and below) should be treated with one of the single agent Methotrexate or Actinomycin D; while high-risk patients (above six scores) will receive multidrug chemotherapy regimens, most commonly EMA-CO (Etoposide, Methotrexate, Actinomycin D, Cyclophosphamide, and Vincristine) [5]. Over 90% of patients can reach complete remission after initial chemotherapy, though there are still some patients who develop recurrent and chemo-refractory tumor after multiple cycles of chemotherapy [6].

The most common metastatic site of choriocarcinoma is lung [7], and other lesions have also been reported before, including brain, adrenal gland, and spleen, manifesting a feature of hematogenous metastasis [8–10]. While intracardiac metastasis is extremely rare, and Ober et al. [11] firstly reported four patients with choriocarcinoma developing intracardiac disease in 100 consecutive patients. To the present, it has reported a total of ten cases of intracardiac metastasis in patients with choriocarcinoma [12–21]. In all reported cases, the sites of cardiac metastasis were diverse, and four cases were cured after surgery combined with chemotherapy or chemotherapy alone.

Herein, it reports a rare case of refractory choriocarcinoma in a patient who developed intracardiac metastases in the right atrium and ventricle after multiple courses of chemotherapy, and underwent resection, but still recurred rapidly after surgery. To the best of our knowledge, this case is the only one in which multiple masses spread throughout the right heart and recurred rapidly after surgery combined with chemotherapy.

### Case presentation

**Fig. 1:**
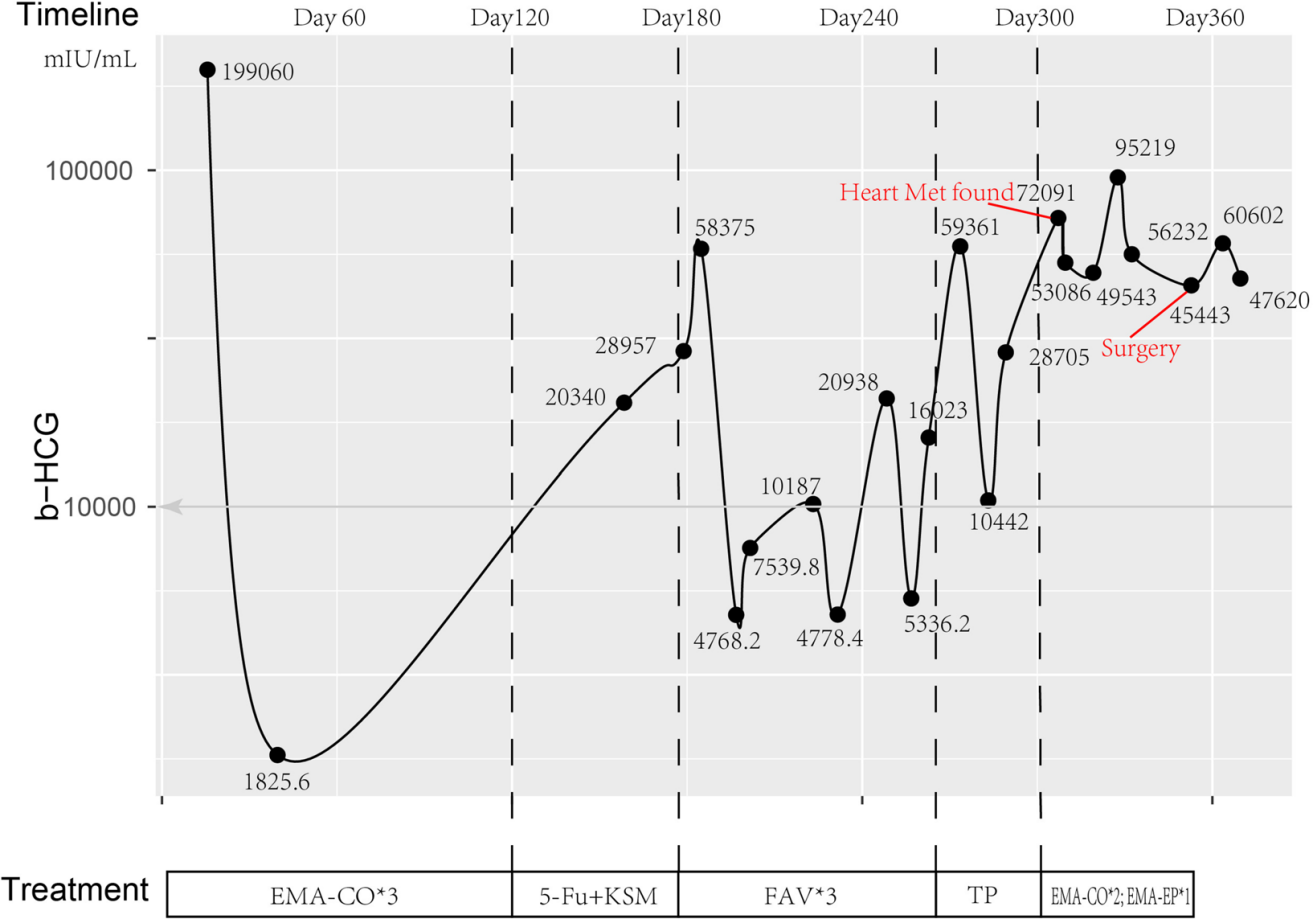
The change of HCG level during chemotherapy

A 37-year-old woman (Gravida 4 Para 3 Abortus 1, and the most recent pregnancy was a normal pregnancy) presented with vaginal bleeding and a high level of β-human chorionic gonadotropin (β-hCG) at 199,060 (mIU/mL). Transvaginal ultrasound showed uneven echogenicity of the endometrium. Chest computed tomography (CT) revealed patchy nodular shadow in bilateral lungs, of which cancer metastasis was suspected, and there was no sign of intracardiac lesion. As a result, the patient was clinically diagnosed with gestational choriocarcinoma (stage III with FIGO score of 12). She initially received standard chemotherapy (etoposide, methotrexate, and dactinomycin with cyclophosphamide and oncovin, EMA-CO*3) but unsatisfactory response was observed, then five cycles of other regimens (5-Fu with Dactinomycin*1; 5-Fu, Dactinomycin and Vincristine, FAV*3; cisplatin and paclitaxel, TP*1) were tried while the level of β-hCG still ranged between 5000 to 10,000 (Fig. 1).

**Fig. 2:**
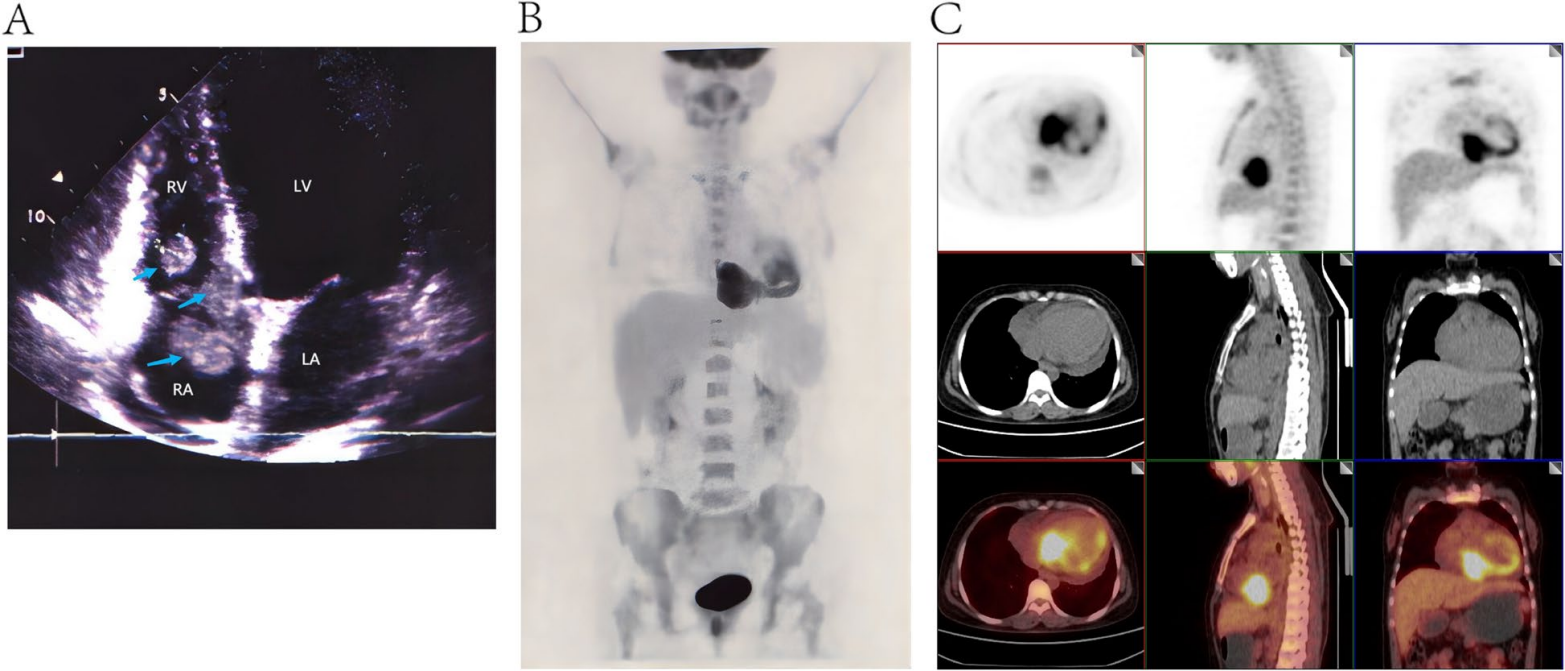
Images of intracardiac metastasis. **A** The image of UCG showed a 6.1*5.4 cm heterogeneous echogenic mass in the right atrium and ventricle, connected with the right atrium and ventricle wall; **B**-**C** The PET/CT scan of the whole body showed malignant mass in the right atrium and ventricle with SUVmax of 9.3

At the time of reexamination after eight courses of chemotherapy, the patient was found intracardiac masses in the right atrium (2.6*1.7 cm), chordae tendineae anterior of the tricuspid valve (1.4*0.7 cm) and the right ventricle (4.1*2.9 cm) by ultrasonic cardiogram (UCG). Then two courses of EMA-CO chemotherapy and one course of EMA-EP (etoposide, methotrexate, and dactinomycin with etoposide and cisplatin) chemotherapy were followed. However, the patient developed a fever with a temperature of 37.5–38.2 ℃, sore throat, chills, chest tightness, dyspnea, and decreased activity tolerance. Moreover, a mid-diastolic grade three murmur can be heard in the tricuspid region on auscultation of the heart. The level of β-hCG climbed to 95,219 (mIU/mL), and UCG revealed a 6.1*5.4 cm heterogeneous echogenic mass in the right atrium and ventricle, connected with the right atrium and ventricle wall (Fig. 2A). Furthermore, positron emission tomography (PET)-CT showed increased irregular metabolic abnormalities in the lower part of the right atrium-right ventricle junction and no other lesion was observed (Fig. 2B-C).

**Fig. 3:**
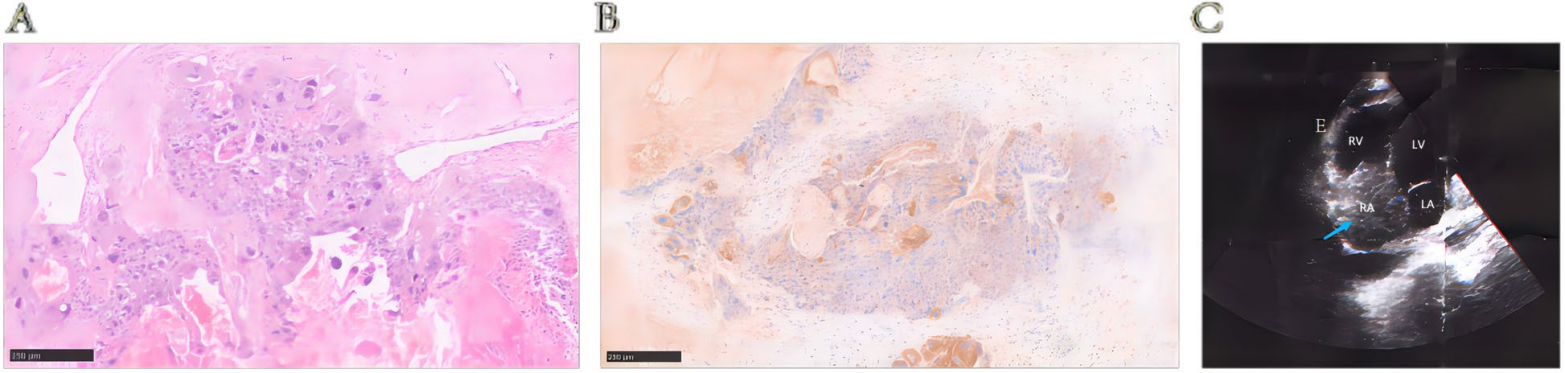
**A** The tissue pathology of the resected mass from right heart (HE, 10 ×); **B** The immunohistochemical staining of β-HCG (positive, 10 ×); **C** A 5.4*4.2 cm mass in the right atrium was found by the UCG after a week of operation

Based on the patient's history of pulmonary metastasis from choriocarcinoma, poor control of β-hCG after multiple courses of chemotherapy, the progressive enlargement of the right heart occupancy (from 2.5*1.4 cm to 6.1*5.4 cm in 2 months), and the occupancy hypermetabolism suggested in PET-CT, the diagnosis of choriocarcinoma with pulmonary and cardiac metastasis was made. After consultation with cardiac surgery, complete intracardiac mass resection was performed, and three intracardiac masses were observed: they were located at the right atrium (3*4 cm), the anterior chordae tendineae of the tricuspid valve (1*2 cm), and the right ventricle (5*7 cm). The pathology confirmed the intracardiac metastasis of gestational choriocarcinoma and the immunohistochemistry showed β-hCG ( +) (Fig. 3A-B). After a week of operation, the UCG found a 5.4*4.2 cm mass in the right atrium again (Fig. 3C). Considering the poor prognosis, the patient received palliative care and eventually died of disease progression.

## Discussion

The occurrence of cardiac metastasis from choriocarcinoma is rare, and the existing literature mainly consists of case reports. Out of the 2300 gestational choriocarcinoma cases observed in the Peking Union Medical College Hospital, only one patient reported heart metastasis. Globally, only 10 cases of cardiac metastases from choriocarcinoma have been reported, which we have summarized in Table 1. Majority of patients have experienced chest discomfort or dyspnea, while gynecological symptoms such as vaginal bleeding were less commonly seen. Notably, a significant percentage (45.5%) of the intracardiac metastatic lesions caused by choriocarcinoma were found in the left atrium [15, 17, 19–21]. And 18.2% of these lesions were located in the right ventricle [16, 18], 18.2% affected blood vessels [12, 14], and one instance where a lesion was found in the left ventricle[13]. And multiple lesions were scattered throughout the right atrium and right ventricle in the case we examined. Further, the current case showed quick recurrence comparing with the other patients, and the potential reason may lie in that the deep invasion of anatomy of heart leaded to limited mass had been resected to reserve the function of heart, and thus, the intracardiac lesion caused by surgery was not smooth which could attract tiny tumor embolus planting, because of heavy load of tumor in our patient. In addition to heart metastases, 54.5% of patients had metastases to other sites, commonly to the lung (83.3%) [12, 19–21], kidney (66.7%) [12, 13, 19, 21], brain (50%) [13, 20, 21], spleen (33%) [13, 21], and gastrointestinal tract (33%) [13, 21]. In some earlier cases, misdiagnosis resulted in poor prognosis and multiple organ metastases at the time of presentation [14, 15]. Unfortunately, in some of these cases, the diagnosis was not confirmed until after the patient's death [12–14, 21].

**Table 1:** Previous cases of intracardiac metastases of gestational choriocarcinoma

	**Status of pregnancy**	**Age**	**Symptoms**	**HCG**	**Cardiac lesion**	**Lesions at other sites**	**Surgical treatment**	**Chemotherapy treatment**	**Immuno-therapy**	**Treatment process**	**Survival**
Akaike A et al	A normal delivery, and a hydatid-form mole	29	A rapid onset of malaise and precordial pain	+ + + (urinary pregnancy test)	Left anterior descending coronary artery	Both lungs and left kidney	TH + BSO; Segmental resection of lung	Methotrexate + Actinomycin-D; Vinblastin per week; Actinomycin-D + Vinblastin	none	The urinary pregnancy test became negative and the disease is in remission many times	Died suddenly 2 years and 11 months after the onset of symptoms
Hepp A et al	Two normal delivery	35	Chest pain, headache and other multi-system symptoms	unknown	Left atrium, caused a high grade stenosis of the mitral orifice	Spleen, intestinal tract, kidneys, pancreas, brain, and pulmonary lymphatics	none	none	none	Rapid deterioration precluded further diagnosis	Died 6 days after admission to hospital
Seigle JM et al	An ectopic pregnancy and a normal delivery	28	headache, nausea, and vomiting	108,000 mIU/ml	Left ventricular apex	Spleen, gastrointestinal tract, kidneys, and brain	none	none	none	-	Died 23 days after initial diagnosis
Vasiljevic JD et al	A normal delivery	26	Metrorrhagia, weakness, and signs of fatigue	increased	Any smaller subepicardial coronary vessels and intra-myocardia	no metastases to any organs or systems	none	Methotrexate + Actinomycin-D	none	Complete remission for 6 months, but soon develop symptoms of heart insufficiency	Died with symptoms of chronic heart failure
Kishore AG et al	Unknown	27	Progressive exertional dyspnea, intermittent fever and loss of weight	High	Left atrium, attached to the interatrial septum	Unknown	Atrial mass excision	Methotrexate + Actinomycin-D	none	Almost certain diagnosis of left atrial myxoma	Died on the 12 postoperative day
Perroni D et al	A normal delivery	22	Chest pain and progressive dyspnea	Unknown	Right ventricle and endocardial	none	Cardiac surgery	Methotrexate + Actinomycin-D; EMA-CO*12	none	Free of disease 4 years and is pregnant again	Remission
Bohlmann MK et al	Three normal delivery	41	Severe dyspnea	782,000 U/L	Left atrium	none	Hysterectomy; Atrial mass excision	EMA-CO*9	none	12 months after cardiac surgery the patient is still in remission	Remission
Bozaci EA et al	Gravida 12 para 10 abortus 2; History of D&C	53	Dyspnea, cough, hemoptysis, and chest pain	200,000 mIU/mL	Right ventricle	none	TAH + BSO	EMA-CO*4 EMA-EP*4	none	Remission for 6 months	Remission
Vincent M et al	Two normal delivery	27	Dyspnea, left chest pain, cough and fever	1032 mIU/mL	Left atrium	Pulmonary and renal	Thrombus excision; Nephrectomy; Pulmonary lesion excision	Cisplatin + etoposide*4	none	Remain disease-free 16 years and give birth to another child	Remission
Li Y et al	A normal delivery	36	Dizziness, headache, and left-side hemiparesis	350,000 mIU/mL	Left atrium	Lung and brain	Unknown	Unknown	Unknown	Unknown	Unknown
Present case	Gravida 4 para 3 abortus 1,	37	Vaginal bleeding	199,060 mIU/mL	Right atrium; Chordae tendinae anterior of tricuspid valve; Right ventricle	Lung	Complete intracardiac masses resection	EMA-CO*5; 5-Fu with KSM*1; FAV*3; TP*1; EMA-EP*1	none	A right atrial mass was found again 1 week after surgery	Died of disease progression

For patients with cardiac metastases or both cardiac and pulmonary metastases, achieving complete remission is possible through a combination of chemotherapy and surgery or chemotherapy alone [16–19]. Therefore, heart metastases from choriocarcinoma are curable, and early diagnosis, chemotherapy, and surgery can lead to a favorable prognosis. When women at childbearing age presenting with a cardiac mass and with a history or clinical symptoms suggestive of trophoblastic disease, it is essential to keep in mind the possibility of cardiac metastases from choriocarcinoma.

Malignant choriocarcinoma cells originating from the primary site by blood circulation occasionally implants in the heart, which causes intracardiac disease [20]. We noted that 45.5% of intracardiac metastatic lesions were found in the left atrium, and that maybe attribute to the special anatomy (left aurcle) and hemodynamics (blood reflux from four lung arteries) in left atrium make tiny tumor cluster easy to implant. Cardiac tumor can be with no symptoms, just nonspecific symptoms, and specific heart-associated symptoms like acute myocardial infarction, heart failure and arrythmias [22]. We also found that majority of patients have experienced chest discomfort or dyspnea likely symptoms of acute left failure, and no significant difference of symptoms was observed in patients with left and right heart metastasis owing to limited cases. The symptom level of cardiac tumor depends on its location, size, growth speed and invasiveness [23]. In some cases, patients without symptoms would be diagnosed when occasional examination or autopsy [24]. Symptomatic patients can be diagnosed by ECG, chest CT scan and PET/CT [25]. Choriocarcinoma patients, with a high proportion of lung metastasis, are regularly received chest CT scan during follow up, which is in favor of recognition of intracardiac lesion [26]. ECG should be considered as the initial examination after revealing intracardiac abnormality to confirm the size, invaded structure, and heart function [24].

The treatment of patients with intracardiac metastasis mainly depends on chemotherapy and surgery [23]. Standard chemotherapy, such as four cycles of EMA/CO and FAEV, should be given to patients to control the primary disease [2]. When a low level of β-hCG has been reached, intracardiac tumor resection should be considered to remove the lesion, and subsequent chemotherapy is recommended for consolidation [26]. In some circumstances, patients can be cured by surgery combined with chemotherapy [22]. In the three patients presenting isolated intracardiac metastasis[16–18], they all showed optimal response to chemotherapy and surgery, which illustrated that GTN with isolated intracardiac metastasis was curable. Intracardiac metastasis is frequently considered as an aggressive sign of disease, it often combines with lung and multiple disease, including brain and spleen. And in these patients, tumor removal is a palliative procedure [22] and the prognosis is unfavorable.

Thought the current case did not received immunotherapy, and there is no report of patients with GTN and intracardiac metastasis who experience PD-1 and PD-L1 inhibitor, immunotherapy in GTN has made great progress in recent years, with successful therapeutic outcomes in several clinical studies [26–29]. Previous researches have revealed that programmed cell death ligand (PD-L1) is highly expressed in GTN tumor tissue [30, 31], and other immune targets TIM-3, LAG-3, and GAL-9 are also widely expressed in GTN [32]. The CAP-01 clinical trial confirmed the efficiency and safety of PD-1 inhibitor in patients with chemo-refractory and relapsed CTN, and showed a 55% of objective response rate [28]. A recent retrospective multicenter study of patients with high-risk chemo-refractory and relapsed GTN showed that PD-1 inhibitor combined with chemotherapy was superior to PD-1 inhibitor monotherapy in real-world settings [27]. Hence, it is to be investigated that GTN patients with intracardiac lesion could benefit from PD-1 immunotherapy combined with chemotherapy.

## Conclusion

Intracardiac metastasis of GTN is an aggressive sign of disease. Patients can benefit from chemotherapy and surgery. Future investigation of PD-1 immunotherapy combines with chemotherapy are expected to improve the prognosis in this group of patients.

## Data Availability

Data are available on reasonable request. The corresponding author could be contacted with requests.

## Abbreviations

GTN: Gestational trophoblastic neoplasia
β-hCG: β-Human chorionic gonadotropin
UCG: Ultrasonic cardiogram
FIGO: The International Federation of Gynecology and Obstetrics
EMA-CO: Etoposide, Methotrexate, Actinomycin D, Cyclophosphamide, and Vincristine
FAV: 5-Fu, Dactinomycin and Vincristine
TP: Cisplatin and paclitaxel
EMA-EP: Etoposide, methotrexate, and dactinomycin with etoposide and cisplatin
PET-CT: Positron emission tomography computed tomography
PD-L1: Programmed cell death ligand-1
